# Unpacking vulnerability to sexually transmitted infections (STIs)/human immunodeficiency virus (HIV) among adolescent girls and young women in India: A qualitative study

**DOI:** 10.1371/journal.pone.0336593

**Published:** 2025-11-24

**Authors:** Sohini Paul, Radhika Dayal, Anupam Joya Sharma, Kuhika Seth, Sowmya Ramesh, Niranjan Saggurti

**Affiliations:** 1 LEAD at KREA University, Chennai, Tamil Nadu, India; 2 Population Council Consulting Pvt Ltd, Delhi, India; 3 Adolescent health Champions, San Francisco, California, United States of America; 4 International AIDS Vaccine Initiative, Delhi, India; 5 Population Council Institute, Delhi, India; 6 Population Council, Delhi, India; Jiangsu provincial Center for Disease Control and Prevention, CHINA

## Abstract

**Background:**

Despite national declines in HIV prevalence, adolescent girls and young women (AGYW) in India remain disproportionately vulnerable to sexually transmitted infections (STIs), including HIV. This vulnerability stems from a convergence of biological, social, and structural factors, including early marriage, gendered expectations, poverty, and limited access to sexual and reproductive health (SRH) information and services. While prior research has documented these determinants, few studies explore how they intersect and evolve to shape AGYW’s lived experiences of risk, particularly within the general population.

**Methods:**

This qualitative study used an adapted socio-ecological framework (which conceptualizes how structural, community, and individual-level factors interact to influence health and vulnerability) to examine the pathways of vulnerability to STIs and HIV among AGYW aged 16–24 in two urban regions: Delhi NCR and Mumbai. Data were collected through 42 in-depth interviews (IDIs), 4 focus group discussions (FGDs), and 18 key informant interviews (KIIs) with health providers, NGO staff, and program implementers. Reflexive thematic analysis was applied, guided by deductive codes from the eco-social model and inductive insights from participants’ narratives.

**Results:**

Findings show that AGYW’s vulnerability is shaped by interconnected macro (e.g., early marriage, patriarchal norms), meso (e.g., school-based silences, provider bias), and micro (e.g., relationship coercion, low self-efficacy) level factors. Although some AGYW had SRH knowledge, stigma, lack of autonomy, and unsupportive environments often constrained its use. Married and low-income AGYW were particularly disadvantaged, while non-governmental organizations (NGOs) played an important but uneven role—offering crucial safe spaces for awareness and support, yet limited by inconsistent coordination and resources.

**Conclusion:**

AGYW’s vulnerability to STIs/HIV in urban India is produced through dynamic and intersecting structural, institutional, and interpersonal constraints. Addressing these requires integrated, gender-sensitive interventions that promote agency, reduce stigma, and foster collaboration between NGOs and public systems. Programs must go beyond awareness to reshape the environments in which AGYW make sexual health decisions.

## Introduction

India has witnessed a commendable decline in national HIV prevalence over the past two decades, with adult prevalence falling from 0.38% in 2001 to an estimated 0.21% in 2021 (National AIDS [1]). However, these overall improvements conceal persistent gendered disparities, especially among adolescent girls and young women (AGYW; aged 16–24 years), who continue to face structural and social barriers to sexual and reproductive health (SRH).

According to national data, only 18.9% of women aged 15–24 possess comprehensive HIV knowledge, and merely 2% of adolescent girls aged 15–19 have ever been tested for HIV [1,2]. These statistics underscore the gap between awareness and access to services, particularly among AGYW from disadvantaged or married groups. By highlighting these figures, we emphasize the continued relevance of examining how structural inequities translate into everyday health risks for young women in urban India.

AGYW face disproportionate risks related to sexually transmitted infections (STIs), including HIV, due to the convergence of biological susceptibility, socio-cultural expectations, systemic inequalities, and limited agency. While numerous studies have identified determinants such as early marriage, poor SRH knowledge, and gender-based violence, far fewer have examined how these factors interact and reinforce one another over time to shape lived experiences of vulnerability. This study addresses the gap by investigating the layered, intersecting, and evolving dynamics of AGYW vulnerability in India, using a qualitative pathways approach grounded in an eco-social framework.

### Defining AGYW in the Indian context

In India, the term AGYW encompasses a diverse and transitional population, ranging from school-going adolescents to married young women, young mothers, and recent migrants. These groups differ in their experiences of mobility, access to education, exposure to SRH information, and autonomy in sexual relationships. Our operational definition of AGYW (ages 16–24) reflects this heterogeneity and focuses on a critical life stage marked by sexual debut, relationship formation, and family pressures around marriage and reproduction. By examining these dynamics, we aim to understand who is vulnerable and *how* and *why* that vulnerability manifests across time and contexts.

### Existing literature and gaps

There is moderate literature demonstrating that AGYW in India experience heightened risk for HIV and other STIs due to early marriage, limited SRH knowledge, gender-based violence, and restricted access to healthcare [3,4,5]. According to the National Family Health Survey (NFHS-5, 2019–21), nearly 23% of women in India are married before the legal age of 18, and 6.8% of adolescent girls have already experienced childbirth. Further, 7.7% of ever-married women aged 15–24 report having experienced sexual violence. Despite legal protections, social norms continue to inhibit agency, rendering AGYW particularly vulnerable in both marital and premarital contexts.

Much of the current research on AGYW vulnerability is fragmented. Quantitative studies often quantify prevalence without adequately capturing the processes through which risk is constructed and experienced [3,6]. While qualitative research offers greater depth, it has disproportionately focused on high-risk populations, such as female sex workers (FSWs) or trafficked girls [7,8], leaving a dearth of insight into the broader population of AGYW. Moreover, many studies analyze vulnerabilities in isolation, focusing on knowledge gaps, service availability, or individual behaviors without examining how these factors intersect to reinforce and sustain systemic risk.

Notably, few studies critically interrogate the institutional and structural stigma that shapes AGYW’s access to SRH services. Bhugra et al. [9] highlight the conservative sexual attitudes that inhibit SRH access but often do not explore how these norms manifest within institutions such as schools and clinics. Likewise, Dayal and Gundi [10] document service delivery failures at adolescent clinics yet stop short of connecting these gaps to underlying structural neglect, provider bias, or policy fragmentation.

In summary, previous qualitative research has primarily centered on high-risk populations—such as female sex workers or trafficked adolescents—thereby overlooking the broader population of AGYW whose vulnerabilities manifest within normative family and community settings. Moreover, prior studies have often treated determinants such as early marriage, SRH knowledge, or service access in isolation. Our study contributes to the literature by examining these factors as interlinked processes—what we term “pathways of vulnerability”—that evolve dynamically across social and institutional levels.

By combining voices of AGYW with those of service providers, NGO staff, and government implementers, this study also bridges the analytical gap between individual experiences and institutional practice. It thereby offers an integrated understanding of how policy intent, program delivery, and lived realities interact to shape AGYW’s vulnerability in urban India.

### A pathways approach to vulnerability

This study advances the literature by focusing on the interconnected *pathways* of vulnerability—how overlapping and interacting determinants cumulatively shape AGYW’s experiences. We adopt a grounded theory approach [11] to analyze how individual, interpersonal, community, and structural factors influence one another across time and space. This perspective allows us to move beyond static listings of barriers to uncover the underlying dynamics and sequences that reinforce risk.

For example, an AGYW may be aware of safe sex practices but unable to apply this knowledge due to stigma from peers, fear of judgment from providers, or lack of autonomy within a relationship. Our study focuses on these relational dynamics, how social norms interact with institutional practices and policy gaps to create compounded vulnerabilities.

This approach is especially relevant in the Indian context, where SRH decisions are not made in isolation but within the constraints of patriarchal family structures, caste hierarchies, economic insecurity, and healthcare gatekeeping [12,13].

### Rationale for the eco-social framework

To unpack these layered vulnerabilities, we draw on the eco-social (socio-ecological) framework by Krieger [14], which conceptualizes how macro-level structures (e.g., laws, norms, policies), meso-level institutions (e.g., schools, health systems, NGOs), and micro-level relationships (e.g., family and partners) interact to influence health outcomes.

This framework is particularly suitable for the Indian context, where AGYW’s sexual health behaviors are embedded in hierarchical social systems shaped by gender, caste, and economic dependency. By situating AGYW’s experiences within this multi-level lens, the framework allows us to trace how social norms, institutional barriers, and individual agency intersect to produce differential vulnerability to STIs and HIV.

### Site selection: Why Delhi NCR and Mumbai?

This study was conducted in two major urban regions: Delhi National Capital Region (NCR) and Mumbai. These locations were purposively selected due to their diverse populations, complex health infrastructures, and status as hubs of internal migration. Delhi NCR includes both urban and peri-urban communities and has seen intensive programming around adolescent health, making it a useful site for evaluating policy reach and effectiveness. Mumbai, India’s financial capital, provides a contrasting setting with its dense slum settlements, high mobility, and stark inequalities in SRH access.

Together, these cities allow us to compare and contrast the vulnerabilities faced by AGYW across socio-economic and spatial divides within urban India—contexts that are often understudied in favor of rural or high-risk settings.

Moreover, we chose the metropolitan cities of New Delhi NCR and Mumbai as the study sites since Maharashtra (ranked first) and Delhi (ranked sixth) were two states with high reports of new HIV infections in 2020 [15]. Approximately 3.90 lakh people in Maharashtra were living with HIV. At the same time, the states of West Bengal, Delhi, Punjab, Rajasthan, Madhya Pradesh, Odisha, and Haryana contributed another 18% of the total PLHIV [16]. At the subnational level, the highest adult HIV prevalence was in the northeastern region. However, Delhi has a prevalence of 0.41%, and Maharashtra has a prevalence of 0.36% among the 15–49-year population [15].

### Contribution of the study

This study makes several key contributions to the existing literature on adolescent sexual and reproductive health in India. First, it offers a dynamic and intersectional understanding of vulnerability among AGYW by analyzing how multiple factors—ranging from structural norms and institutional barriers to interpersonal relationships and individual knowledge—interact to shape health outcomes. Unlike prior research that often examines these determinants in isolation, our study traces the *pathways* through which vulnerabilities accumulate and reinforce one another. Second, by focusing on AGYW from the general population rather than solely on high-risk groups such as sex workers or trafficking survivors, this study broadens the scope of inquiry and highlights overlooked experiences within everyday urban contexts. Third, the study critiques existing policies, such as the Rashtriya Kishor Swasthya Karyakram (RKSK), by examining how implementation gaps—due to stigma, poor institutional coordination, and inadequate outreach—limit their effectiveness. Fourth, our research includes perspectives from multiple stakeholders, including AGYW, healthcare providers, and NGO workers, allowing for a holistic view of both service delivery and demand-side constraints. Finally, through a comparative lens across two major urban centers—Delhi NCR and Mumbai—this study captures spatial and socio-economic variations in vulnerability, offering insights that can inform more context-specific and integrated adolescent health interventions. In doing so, it responds to critical gaps in both policy and research, proposing a more nuanced and comprehensive framework for addressing AGYW’s vulnerability to STIs and HIV in India.

## Methods

### Research design

This study employed a qualitative research design grounded in constructivist grounded theory [17] to explore the pathways of vulnerability to STIs and HIV among adolescent girls and young women (AGYW) in Delhi NCR and Mumbai. A qualitative approach was appropriate given the exploratory and context-sensitive nature of our research question. AGYW’s sexual and reproductive health (SRH) vulnerabilities are shaped by deeply embedded cultural, structural, and interpersonal dynamics that quantitative approaches often overlook [18].

**Fig 1:**
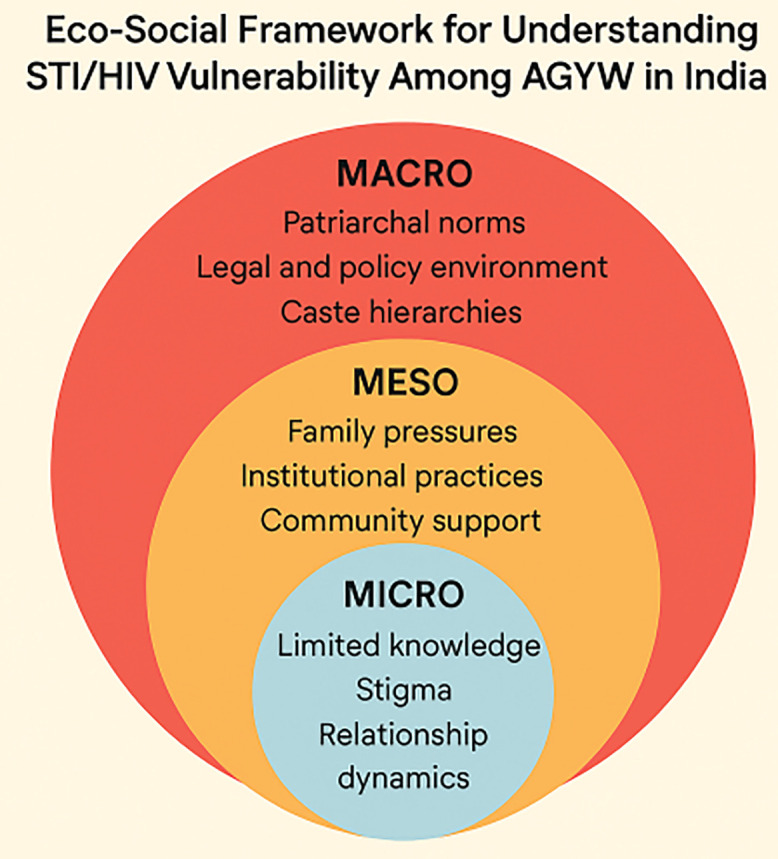
Adapted Eco-Social Framework for Understanding AGYW Vulnerability to STIs/HIV.

We used the eco-social framework [14] as our analytic scaffold (Figure 1). This framework conceptualizes how social, institutional, and structural determinants interact over time to influence health outcomes, enabling us to organize data into macro, meso, and micro layers of vulnerability. The figure illustrates the interaction of three interrelated levels influencing adolescent girls’ and young women’s (AGYW) vulnerability: macro-level structural factors (e.g., gender norms, policy gaps, poverty); meso-level institutional and community factors (e.g., school-based silences, healthcare bias, NGO interventions); and micro-level individual and interpersonal factors (e.g., self-efficacy, partner negotiation, stigma). Arrows depict the dynamic and bidirectional relationships among levels, emphasizing that vulnerability emerges through cumulative and intersecting processes rather than isolated determinants. The framework guided both the coding process and the interpretation of themes presented in the Results section.

### Sampling and participants

We used purposive sampling [19] to ensure variation in age, marital status, education, social class, and sexual experience. Forty-Two AGYW aged 16–24 participated in in-depth interviews (IDIs), complemented by four focus group discussions (FGDs). Participants were recruited from low-income and more privileged backgrounds to represent diverse urban experiences.

To supplement purposive sampling and enhance diversity, we used snowball sampling [20], especially to recruit AGYW from higher education institutions who were harder to reach through community-based organizations. Although non-probability sampling techniques may inherently introduce selection bias, we undertook several measures to minimize its impact. We strategically diversified our recruitment sites to include a broad range of settings—such as community centers, health clinics, educational institutions, and informal gathering spaces—to capture varied socio-demographic and geographic contexts. Additionally, we prioritized the inclusion of participants from diverse backgrounds in terms of age, socioeconomic status, education levels, and sexual and reproductive health experiences. This intentional heterogeneity enhanced the representativeness of our sample and strengthened the credibility and transferability of our qualitative findings. The manuscript is aligned with the COREQ checklist [21].

### Key informants (KIs)

We conducted 18 Key Informant Interviews (KIIs) with individuals who had programmatic, clinical, or community experience in adolescent health. These included: Clinical doctors and nurses from both public and private hospitals; NGO program managers, peer educators, and field workers; Government health officials involved in RKSK implementation.

KIIs were used to understand service-level constraints, policy implementation challenges, and systemic stigma. Unlike IDIs and FGDs with AGYW that explored lived experiences, KIIs provided institutional and structural perspectives, complementing and contextualizing AGYW narratives [22].

### Data collection

We collected data during March and September 2023. Two trained female researchers experienced in SRH qualitative research conducted interviews and FGDs. All sessions took place in private, safe locations: NGO offices, health centers, or mutually agreed-upon spaces. Interviews lasted 40–90 minutes on average.

For AGYW under 18, parental consent and adolescent assent and for adult participants, informed consents were obtained in line with ethical protocols [23]. Sessions were audio-recorded with participants’ permission and transcribed verbatim. All transcripts were translated into English where necessary.

To build trust, interviewers began with general rapport-building questions and gradually transitioned to sensitive topics. Participants could opt to go off-record at any point or pause interviews when needed. We also provided referral resources for psychological support if needed. These strategies helped minimize psychological distress and ensured emotional safety [24].

### Reflexivity and researcher positionality

The research team engaged in continuous reflexivity. Interviewers were women with strong experience in qualitative research involving AGYW and backgrounds in public health and sociology.

women interviewers deliberately positioned themselves as empathetic listeners rather than authoritative figures. To reduce interviewer influence on participant responses, they consistently used open-ended questions, maintained neutral body language, and provided repeated reassurances about confidentiality throughout the conversations.

Additionally, daily debriefing sessions were conducted among the research team to reflect on fieldwork experiences, discuss challenges encountered, process emotional responses, and identify any emerging biases that might affect data collection or interpretation. We also maintained writing memos and positionality journals. This helped recalibrate the interview approach and maintain sensitivity to participants’ social contexts [25].

### Trustworthiness and data saturation

To enhance credibility, we employed triangulation across: Methods (IDIs, FGDs, KIIs), data sources (AGYW and professionals) and locations (Delhi NCR and Mumbai). Member checks were conducted with a subset of AGYW to validate interpretations. We monitored data saturation during concurrent analysis and determined it had been reached when no new codes or themes emerged from subsequent interviews [26].

### Ethical considerations

Ethical approval was obtained from the Sigma Scientific Board (10071/IRB/22–23). Informed consent and assent were taken, and participants were informed of their right to withdraw at any time. All the consents were collected in written format, where anonymity and confidentiality clauses were explicitly declared. All personal identifiers were removed, and pseudonyms were used to maintain confidentiality.

Researchers were trained to monitor emotional discomfort and were prepared to refer participants to trusted counselors or NGO staff if required. However, no severe distress was observed during fieldwork.

### Data analysis

We used reflexive thematic analysis [27,28], supported by NVivo 12. This qualitative approach was chosen because it allows exploration of how and why vulnerabilities are experienced and constructed—insights that would not be attainable through quantitative designs alone. While quantitative data can identify the prevalence of early marriage or limited service access, qualitative inquiry reveals the lived meanings, emotional consequences, and contextual power dynamics that shape these experiences. For instance, participants’ narratives exposed subtle forms of relational coercion and institutional stigma that numerical indicators could not capture.

Seven representative transcripts were initially open-coded. Coding was conducted independently by three researchers and then refined through iterative team discussions to build a comprehensive coding tree. We adopted a hybrid approach combining deductive and inductive strategies: deductive codes were derived from the eco-social framework (e.g., “macro-level norms,” “meso-institutional barriers,” “micro-relationship power”), while inductive codes emerged from participants’ phrasing and recurring concepts (e.g., “school silence,” “fear of judgment,” “negotiating protection”).

To ensure transparency and analytic rigor, each code was linked to verbatim quotations in NVivo. Codes were then grouped into preliminary categories (e.g., “sources of SRH knowledge,” “gendered decision-making,” “provider bias”). Through constant comparison and memo-writing, these categories were progressively refined into overarching themes. For example, the initial codes “teacher avoidance,” “embarrassment discussing menstruation,” and “skipped chapters” were merged into the theme “Institutional silences in SRH education.” Similarly, “partner resistance,” “emotional pressure,” and “condom negotiation” informed the theme “Gendered autonomy and relational coercion.”

Themes were elaborated through: (i) theme merging—when overlapping codes were identified; (ii) theme refinement—by revisiting and collapsing subcategories; and (iii) tri-level mapping—aligning themes with macro, meso, and micro layers of the eco-social model. The final thematic map, validated against our code matrix (see Supplementary Codesheet in S1 Data), comprised three central themes: (1) SRH knowledge and misinformation; (2) sexual autonomy and partner negotiation; and (3) health-seeking behaviors and stigma. These reflected the dynamic interactions among structural, institutional, and individual-level forces shaping AGYW vulnerability. Each theme was mapped against the corresponding level of the eco-social model (see Fig 1), allowing us to trace how structural, institutional, and interpersonal forces converged to shape AGYW’s lived experiences.

### COREQ compliance

This study adheres to the Consolidated Criteria for Reporting Qualitative Research (COREQ) 32-item checklist [21]. We transparently report researcher characteristics, sampling strategies, interview settings, data analysis processes, and ethical safeguards. A completed COREQ checklist is available in the supplementary materials.

## Results

### Overview of sample characteristics and analytical strategy

Our study included 42 in-depth interviews (IDIs), 4 focus group discussions (FGDs), and 18 key informant interviews (KIIs) across two urban locations: Delhi and Mumbai. Table 1 outlines the socio-demographic composition of our AGYW sample. While the majority had completed secondary school, notable variation existed by city and marital status: Mumbai had a higher proportion of married AGYW (20%) compared to Delhi (12.5%), and a greater proportion of participants engaged in paid work (14.3% vs. 3.13%). Though statistically insignificant, these indicative differences informed our intersectional reading of qualitative narratives, allowing us to understand how economic status, marital status, and geography shaped AGYW’s awareness, autonomy, and health-seeking behavior.

**Table 1:** Socio-economic status of the participants.

Characteristic (%)	Mumbai N = 35 (IDI- 22; FGD-13)	Delhi N = 32 (IDI- 20; FGD- 12)
**Age**		
15-17 years	5.71	9.38
18-24 years	94.29	90.63
**Education**		
Primary	2.86	
Middle school	22.86	15.63
High school	11.43	15.63
Secondary school	34.29	62.50
Graduate	14.29	6.25
Pursuing Masters	14.29	0.00
**Currently working**		
Yes	14.29	3.13
No	82.86	96.88
**Monthly household income (INR)**		
0-10000	14.29	28.13
10000-25000	28.57	28.13
25000-50000	22.86	9.38
50000 and above	25.71	28.13
Do not know	11.43	6.25
**Religion**		
Hindu	60.00	75.00
Muslim	25.71	18.75
Christian	5.71	3.13
Buddhist	8.57	0.00
Do not know/Not responded	0.00	3.13
**Marital status**		
Married	20.00	12.50
Unmarried	80.00	87.50
**Current relationship status**		
Single	45.71	56.25
In a relationship	34.29	31.25
Did not share/NA	2.86	3.13
**Currently pregnant**		
Yes	11.43	3.13
No	88.57	96.88
**Currently breastfeeding**		
Yes	5.71	3.13
No	94.29	96.88

*Note: Author’s calculation*

Using the adapted eco-social model (Fig 1), we categorized findings across macro- (structural/policy), meso- (institutional/community), and micro- (individual/interpersonal) levels. The results are presented under three broad thematic categories: (1) Awareness and Knowledge of SRH, (2) Sexual Autonomy and Negotiation, and (3) Health-Seeking Behaviors. Each theme integrates qualitative insights with quantitative patterns from Table 1 to reveal key relationships and contrasts across sub-groups ensuring consistency between the conceptual framework and thematic analysis.

### Theme 1: Awareness and knowledge of SRH, STIs, and HIV

#### Mixed levels of awareness: Stratified by education, marital status, and city.

AGYW’s awareness of sexual and reproductive health (SRH) and HIV varied markedly by education, marital status, and city. Unmarried AGYW pursuing higher education in Mumbai reported greater exposure to formal SRH information through college curricula, peer networks, or digital platforms. In contrast, married adolescents and those living in Delhi’s low-income neighborhoods reported little or no formal instruction. As one Delhi respondent explained, *“I heard about HIV only after my cousin got married and became sick. Before that, we never talked about it.”*

Even among educated participants, understanding was inconsistent. A graduate from Delhi accurately described HIV transmission and preventive practices, while another with similar education confused HIV with casual contact and unclean toilets—revealing that knowledge was often fragmented and context-dependent. Married AGYW from Mumbai’s underprivileged communities frequently equated condom use only with pregnancy prevention. As one young woman said, *“I just know that using a condom prevents pregnancy,”* unaware of its role in preventing STIs. These gaps show how awareness depends not only on education but also on access to safe and accurate information spaces.

Cross-city comparisons from our coded data indicated that Mumbai participants—particularly those with secondary or higher education—referenced NGO-led workshops and online platforms more frequently than their Delhi counterparts, suggesting stronger integration of institutional and digital information channels.

In Delhi, awareness was more often shaped by informal peer discussions or fragmented school instruction, reflecting weaker NGO presence and more conservative community environments.

Educational attainment strongly intersected with marital status: higher-educated unmarried AGYW displayed greater knowledge diversity, while married participants, regardless of schooling, reported limited exposure and lower confidence discussing SRH.

Coding frequencies also showed that references to “condom negotiation” and “HIV prevention” were nearly twice as common among Mumbai respondents as among those from Delhi, underscoring city-level variations in exposure and empowerment. Collectively, these cross-sectional patterns highlight how urban context, education, and marital status interact to shape both the depth and accuracy of SRH knowledge among AGYW.

Interpreted through the eco-social framework, these patterns reveal how structural forces (macro level, such as education and urban opportunity), institutional contexts (meso level, including NGO and school systems), and interpersonal settings (micro level, such as family control and peer influence) jointly determine SRH awareness. For instance, the greater availability of NGO programs in Mumbai illustrates meso-level institutional support mitigating macro-level gender norms, while the silences within Delhi families show how micro-level dynamics reinforce structural barriers. This multilevel interpretation clarifies that awareness is not a single variable but an outcome produced through interactions across social systems.

#### Sources of information: Influence of schools, families, and NGOs.

Schools were the earliest formal setting for SRH education, yet delivery was inconsistent. Several participants recounted that male teachers skipped reproductive-health chapters or discussed them hastily, while college environments encouraged more open, reflective conversations—particularly among economically advantaged girls.

Families were generally silent on SRH issues, and fear of judgment or punishment—especially among girls from conservative or low-income households—limited discussion. A 19-year-old from Mumbai remarked, *“If my parents found out I had a boyfriend, they would stop my education.”* Married AGYW were almost entirely excluded from family-level SRH communication.

NGOs filled crucial gaps by creating youth-friendly, non-judgmental spaces that offered awareness on contraception, STIs, and inclusive discussions about relationships. Despite cultural resistance and limited institutional coordination, many organizations successfully built AGYW trust by embedding SRH content within broader life-skills or empowerment programs. These spaces often provided the only consistent and reliable information for both married and unmarried girls.

#### Synthesis with quantitative patterns (Table 1).

Patterns from Table 1 reinforced these qualitative findings. AGYW in Mumbai—where NGO engagement and employment opportunities were higher—showed greater SRH awareness than peers in Delhi. Lower awareness among married participants reflected how early marriage curtailed educational continuity and restricted access to credible SRH information.

### Theme 2: Sexual autonomy and negotiation of safe practices

#### Constraints of early marriage and patriarchal norms.

Across Delhi and Mumbai, early marriage emerged as a critical macro-level constraint on AGYW’s sexual autonomy and broader well-being. Participants described marriages arranged as early as 14 or 15 years of age, often driven by economic hardship, entrenched patriarchal expectations, and social imperatives to preserve family honor. Once married, young women—especially those from low-income households with limited education—lost what little autonomy they previously held, with negligible control over contraceptive choices, sexual initiation, or pregnancy timing. Accounts of intimate partner violence underscored how marriage, idealized as protective, often institutionalized control over women’s bodies and reinforced dependency. A 16-year-old from Delhi, married to an older man, described being denied food and physically assaulted during pregnancy—illustrating the embodied consequences of gendered power and deprivation. Quantitative findings supported these intersections: 83% of married AGYW lived in low-income households and had completed only primary education. Compared to unmarried peers in Mumbai with higher education, whose exposure to SRH discourse was greater, married adolescents in Delhi were structurally and socially constrained. The eco-social framework helps contextualize these experiences as outcomes of macro-level forces—economic precarity, patriarchal family systems, and early marriage norms—interacting to shape micro-level vulnerabilities.

#### Negotiation in casual relationships: A mixed picture.

In contrast, unmarried AGYW—particularly college-going women in Mumbai—described greater exposure to digital and social spaces that facilitated non-marital or casual relationships. Yet, higher education and urban residence did not guarantee sexual agency. Several participants across both cities described male partners resisting condom use or employing emotional manipulation, revealing persistent relational inequities. As one Delhi participant recounted, “He forced me so much. I told him if he wanted to do it, it had to be with protection. But he said no.” These narratives challenge assumptions that urban or educated women are inherently empowered; rather, autonomy was contingent, negotiated within unequal emotional and gendered power structures. From an eco-social lens, this reflects the meso-level interplay between relational norms, emotional dependencies, and peer influences, which mediate how broader gender ideologies manifest in intimate encounters.

#### Emerging enablers: Peer support and NGO mediation.

Amidst these structural and relational constraints, some AGYW demonstrated agency through the enabling influence of NGOs and peer networks. Peer educators and NGO-led interventions—more accessible in Delhi’s community-based settings than in Mumbai’s college networks—provided safe spaces for dialogue, knowledge-sharing, and modeling of assertive negotiation behaviors. A former FSW in Delhi recalled refusing clients who declined condom use, emphasizing how collective reinforcement from peer educators reshaped her confidence and boundaries. These experiences illustrate that sexual autonomy can be cultivated rather than presumed, through sustained engagement and social learning. From the eco-social perspective, these meso-level interventions counteracted macro-level norms by strengthening proximal capacities for agency—knowledge, self-efficacy, and peer solidarity.

#### Cross-level interplay of factors.

Overall, the findings reveal a layered ecology of constraint and resistance: macro-level structures (patriarchal norms, early marriage, economic deprivation) intersect with meso-level influences (NGO engagement, peer networks) and micro-level experiences (relationship dynamics, self-perception). Interventions situated at the meso-level can thus create localized “pockets of resilience,” even in contexts where broader gender hierarchies persist. However, the analysis also cautions that enhancing knowledge alone is insufficient—without addressing the relational and emotional underpinnings of agency, structural inequities will continue to reproduce vulnerability. The eco-social framework underscores that meaningful empowerment of AGYW must simultaneously engage all ecological layers—structural, social, and individual—to enable durable sexual autonomy.

### Theme 3: Health-seeking behaviors and barriers to care

#### Stigma and judgment in healthcare settings.

Across Delhi and Mumbai, pervasive stigma surrounding adolescent sexuality emerged as a key **micro- and meso-level** deterrent to health-seeking behavior. Fear of being labeled “immoral” or “promiscuous” discouraged many AGYW—especially unmarried and low-income young women—from seeking timely sexual and reproductive health (SRH) care. In one Mumbai case shared by a community health worker, a girl delayed seeking treatment for STI symptoms out of fear of exposure, later receiving an HIV diagnosis. Such accounts illustrate how stigma transforms manageable conditions into life-altering crises.

Both IDIs and FGDs revealed that AGYW across socioeconomic groups encountered moralistic or dismissive treatment from healthcare providers, particularly in public facilities. While unmarried adolescents faced heightened suspicion, married AGYW were not exempt; several from Delhi described being “scolded” or “blamed” during antenatal or post-pregnancy visits. These interactions eroded trust and created a cycle of avoidance and delayed care. Stigma was most acutely experienced by poorer and less-educated AGYW, revealing how class- and gender-based prejudices intersect to shape clinical encounters. Through an eco-social lens, such stigma reflects macro-level gender ideologies that regulate female sexuality, meso-level institutional norms within the health system, and micro-level experiences of shame and silence that jointly restrict AGYW’s access to care.

#### Awareness and Its role in health-seeking confidence.

Where community or NGO programs had invested in SRH awareness, AGYW—especially unmarried college-going women in Mumbai and NGO-linked participants in Delhi—reported higher confidence navigating health services. “Now if something happens, at least I know where to go. Before, I would have been too scared to ask anyone,” said one participant from Mumbai. These interventions combined information with emotional safety, offering confidential spaces where girls could ask questions without fear of judgment. Peer and community engagement fostered a sense of solidarity that translated into practical self-efficacy. Within the eco-social framework, these **meso-level enabling environments** demonstrate how social support and shared learning can buffer the effects of structural stigma and empower AGYW to seek timely care.

#### Disconnect between awareness and action.

However, awareness alone did not consistently lead to care-seeking or protective behaviors. Even informed AGYW often refrained from testing or treatment due to persistent macro-level stigma and meso-level provider bias. Fear of confidentiality breaches or gossip within clinics—especially acute in Delhi’s densely connected low-income settlements—discouraged many from acting on their knowledge. This knowledge-action gap underscores that while information is a necessary precondition, it is insufficient without supportive systems and relational safety. The eco-social model clarifies this disjuncture: individual-level understanding can be nullified by unsympathetic institutional practices and normative pressures at higher ecological levels.

#### Underexplored role of government programs.

Despite the presence of adolescent health initiatives such as the Rashtriya Kishor Swasthya Karyakram (RKSK), engagement remained minimal across both cities. Few AGYW recognized or accessed government-run services, citing the absence of outreach and intimidating institutional environments. Key Informants confirmed that limited inter-sectoral coordination, weak community interface, and low visibility of Adolescent Friendly Health Clinics (AFHCs) restricted program reach. In contrast, NGOs operating in urban slums or college settings were more successful in connecting with AGYW due to their sustained presence and trust. This comparison highlights how macro-level policy structures fail to reach the intended population without meso-level mediation by trusted actors. Strengthening collaborations between RKSK and community-based NGOs could bridge this gap, aligning top-down policy intent with bottom-up social credibility.

#### Cross-level synthesis.

Taken together, the findings illustrate an interlocking ecology of barriers and enablers. Macro-level stigma and policy fragmentation, meso-level provider attitudes and institutional weaknesses, and micro-level fear, shame, and limited agency interact to shape AGYW’s health-seeking trajectories. Yet, where meso-level supports—such as peer education and NGO facilitation—were strong, they generated tangible improvements in confidence and access. The eco-social framework thus underscores that sustainable change demands interventions across all layers: structural reforms to reduce stigma, institutional investments in youth-friendly care, and relational spaces that nurture AGYW’s agency and trust in the health system.

## Discussion

This study examined the multi-layered pathways of vulnerability to STIs and HIV among adolescent girls and young women (AGYW) in two major urban centers in India—Delhi NCR and Mumbai. Using an adapted eco-social framework, we analyzed how macro, meso, and micro-level factors interact to shape AGYW’s experiences, focusing on structural constraints, relational dynamics, and institutional gaps.

At the macro level, entrenched gender norms, early marriage, and inadequate policy implementation emerged as significant drivers of vulnerability. Our findings corroborate earlier research showing that early marriage increases health risks and reduces sexual autonomy [3]. Yet, our data show that the impact of such norms is not merely linear but compounded by economic precarity, caste dynamics, and spatial constraints. For instance, low-income married adolescents in Mumbai lacked mobility and awareness, amplifying their risk exposure. This suggests the need to treat marriage not as a singular event but as a gateway into a matrix of vulnerability reinforced by socio-cultural expectations and economic dependence.

Our findings indicate that material deprivation restricts AGYW’s ability to access SRH information and services. Girls from lower-income households were more likely to discontinue education, marry early, and report low awareness of STI prevention. Financial precarity not only reduced access to care but also influenced sexual decision-making—for instance, in the form of transactional relationships or coerced condomless sex. Poverty thus must be considered a structural determinant of SRH vulnerability, not just a background variable. The intersection of caste and class further compounds this precarity: marginalized-caste AGYW often reported discrimination within health facilities and reduced access to NGO programs, underscoring the need for social inclusion strategies in SRH interventions.

Further, parental engagement—or its absence—emerged as a critical meso-level factor. While families often acted as gatekeepers, enforcing silence around SRH, they were also potential enablers. Parents’ discomfort with adolescent sexuality led to control tactics that inadvertently pushed AGYW towards misinformation via peers or social media. Programs must therefore consider how to support parents in engaging constructively with their daughters’ sexual health journeys.

At the meso level, schools, healthcare systems, and NGOs played contrasting roles. Schools often skipped sex education content, especially when teachers were male, perpetuating a culture of silence. In contrast, NGOs provided safe, youth-friendly spaces for dialogue, even among married AGYW. Yet, these organizations often lacked institutional support, and their outreach was fragmented. Notably, peer educator models—though promising—suffered from high attrition and weak links to formal health systems. A concrete pathway for improvement would be to embed NGO peer educators within the RKSK structure through joint capacity-building modules, shared monitoring dashboards, and formal referral protocols connecting NGO outreach to Adolescent Friendly Health Clinics (AFHCs). Such institutionalized linkages could transform NGO efforts from peripheral to integral components of public health delivery.

A major gap in SRH programming is the under-discussion of how deeply gender norms shape AGYW’s autonomy. Across marital and non-marital relationships, AGYW reported coercive dynamics: married participants described sexual violence and lack of contraceptive control, while unmarried respondents often encountered emotional manipulation around condom use. These experiences point to the need to explicitly address gendered power imbalances in all SRH programming. Building autonomy is not merely about knowledge dissemination but also requires interventions that shift interpersonal and societal norms. Comparable studies from Kenya and South Africa have shown that integrating gender-transformative approaches—such as couple communication training and community dialogues—can substantially improve young women’s negotiation capacity and reduce sexual coercion [29,30]. Similar approaches could be adapted for Indian urban contexts through collaboration between community-based organizations and RKSK’s existing adolescent health platforms.

Our urban lens revealed important spatial nuances. While urban areas are typically assumed to have better SRH access, our findings complicate this assumption. Urban poor adolescents—especially those in informal settlements—faced barriers similar to or worse than their rural counterparts, including limited mobility, overcrowded households, and stigma at public clinics. Moreover, Mumbai and Delhi showed variation in AGYW’s SRH knowledge, with Mumbai participants reporting more peer and NGO exposure. These insights point to the need for context-specific, rather than urban-rural binary, interventions. Comparable urban disparities have been documented in Nairobi and Manila, where socioeconomic inequality within cities leads to uneven SRH access despite urban concentration of services [31,32].

While our study centered AGYW’s voices, their narratives often revealed the controlling or enabling roles of male figures—partners who refused condoms, teachers who skipped SRH content, or fathers who surveilled daughters’ movements. Male partners significantly influenced sexual decision-making; AGYW who attempted to negotiate protection were often overridden by partner resistance. This calls for programs that engage men and boys, not only to foster supportive behaviors but also to challenge harmful masculinities and reproductive coercion.

The discussion of intersectionality was further strengthened to capture overlapping axes of disadvantage. Lower-caste girls in Mumbai’s informal settlements faced layered stigma: they were hyper-visible in public spaces and simultaneously silenced in private spheres. Girls with disabilities or those from religious minorities encountered unique challenges in accessing health information and safe services. Intersectional programming must recognize these compounded risks and avoid one-size-fits-all solutions.

Finally, while we acknowledged NGOs as key stakeholders, this revision provides clearer implementation pathways for their integration with public systems such as RKSK. Government stakeholders themselves recognized in key informant interviews that RKSK’s implementation suffered from fragmentation, limited youth engagement, and poor monitoring. Rather than treating NGOs as adjuncts, future frameworks should institutionalize their role through memoranda of understanding (MoUs) at the district level, co-branded IEC materials, joint outreach campaigns, and performance-linked funding models. A clearer governance mechanism for NGO–government collaboration could enhance continuity, accountability, and trust, ensuring that community-based innovations are scaled through the public system.

### Policy and programmatic implications

To summarize, our findings point to the following actionable policy implications:

*Designing multi-level interventions with structural sensitivity*

Programs must address macro norms (e.g., gender inequality, child marriage), meso institutions (e.g., school-based SRH curricula, health service stigma), and micro behaviors (e.g., condom negotiation, STI testing). This requires intersectoral collaboration among education, health, and women’s development departments. Structural levers, such as conditional cash transfers for girls’ education, urban livelihood schemes for young women, and parental counseling modules, must be mainstreamed into SRH strategies.

*Expanding and localizing comprehensive sexuality education (CSE)*

CSE must extend beyond schools to colleges, vocational training centers, and community platforms. Given the reluctance of some male teachers to teach SRH, dedicated SRH facilitators—preferably female and trained in adolescent psychology—should be deployed. Curricula should include discussions on gender norms, consent, pleasure, and healthy relationships, and be adapted for cultural and linguistic contexts.

*Investing in gender-sensitive healthcare training*

SRH modules for providers should go beyond biomedical knowledge and include empathy, confidentiality, and bias mitigation. Youth-friendly health certification should be mandatory for clinics in urban slums and high-prevalence zones. Feedback loops, e.g., anonymous patient satisfaction tools, could help track and improve adolescent-friendly services.

*Strengthening NGO-government integration through institutional frameworks*

We recommend formal partnerships between RKSK and NGOs, with defined roles in awareness generation, service delivery, and follow-up care. Peer educator programs should be funded as part of RKSK with proper training, incentives, and retention strategies. Public-private collaboration platforms may be established to coordinate efforts, share data, and ensure quality assurance.

*Creating safe and inclusive platforms for AGYW*

Peer-led digital platforms, moderated chat services, and anonymous helplines can enable confidential SRH queries. Community safe spaces like youth hubs or adolescent-friendly clubs should also include disabled, married, LGBTQ + , and out-of-school AGYW. Intersectionality may be mainstreamed in program design: mobile SRH clinics for low-mobility groups, sign-language-enabled IEC tools for the hearing-impaired, and culturally adapted messaging for marginalized communities.

*Engaging male stakeholders across touchpoints*

Male educators, peer mentors, partners, and community leaders should be sensitized to adolescent girls’ SRH needs. Interventions should challenge norms around masculinity, consent, and protection use. School-based sessions and social media campaigns targeted at boys can help foster supportive behaviors and reduce stigma.

## Conclusion

This study explores the complex and interconnected factors that contribute to adolescent girls’ and young women’s vulnerability to STIs and HIV in India. Drawing on the eco-social framework, we demonstrate how structural conditions—such as poverty, early marriage, and gender norms—interact with institutional gaps and interpersonal dynamics to limit AGYW’s access to sexual and reproductive health (SRH) information, services, and autonomy. Despite varying awareness levels, most AGYW faced significant barriers to negotiating protection, seeking healthcare, or acting on their SRH knowledge, particularly due to stigma, coercive relationships, and judgmental service environments. Our findings underscore that vulnerability is not merely a function of individual behavior but a product of systemic inequities that require comprehensive and multi-level responses.

To address these vulnerabilities, programs must move beyond awareness generation to actively dismantle structural and relational constraints that AGYW face. This includes investing in gender-sensitive healthcare systems, strengthening NGO-government collaborations, engaging male stakeholders, and designing interventions that are intersectional and responsive to local contexts.

Policymakers may prioritize integrated adolescent health policies that align education, health, and gender ministries; educators must ensure comprehensive sexuality education is inclusive and age-appropriate; and NGO leaders can play a bridging role by linking community trust with institutional accountability. Coordinated action among these stakeholders is essential to transform fragmented efforts into a cohesive ecosystem that enables AGYW’s autonomy and well-being.

Future research should extend this work to rural and peri-urban settings and adopt mixed-method approaches to capture the scale and texture of AGYW’s experiences. Ultimately, promoting AGYW’s sexual and reproductive rights requires not just services, but sustained efforts to shift the social and institutional environments that shape their lives.

## Supporting information

S1 Data COREQ Checklist AGYW Study. (DOCX)
