# Supplementary material for: Unpacking vulnerability to sexually transmitted infections (STIs)/human immunodeficiency virus (HIV) among adolescent girls and young women in India: A qualitative study
Source: PLoS One. 2025 Nov 24;20(11):e0336593. doi: 10.1371/journal.pone.0336593 (PMC12643274; doi:10.1371/journal.pone.0336593)
Supplement: S1 Data — (DOCX) [file pone.0336593.s001.docx]

# Appendix 1: COREQ Checklist (Consolidated Criteria for Reporting Qualitative Research)

Tong, Sainsbury, & Craig, 2007 – 32-Item Checklist

## Domain 1: Research Team and Reflexivity

### Personal Characteristics

- Interviewer/facilitator:

All interviews and FGDs were conducted by trained qualitative researchers, primarily female, with experience in SRH research.

- Credentials:

Team included researchers with MA, MPH, and PhD degrees.

- Occupation:

Researchers were affiliated with academic institutions or public health NGOs / for-profits at the time of the study.

- Gender:

The primary data collectors were female.

- Experience and training:

Team members were trained in qualitative methods and underwent project-specific training in ethics and trauma-informed interviewing.

### Relationship with Participants

- Relationship established:

No prior relationships existed between researchers and participants.

- Participant knowledge of interviewer:

Participants were informed of the researchers’ affiliations and study objectives.

- Interviewer characteristics:

Researchers shared positionality (gender, caste/class) and reflected on potential influence during analysis.

## Domain 2: Study Design

### Theoretical Framework

- Methodological orientation:

Constructivist grounded theory guided the study design and analysis.

### Participant Selection

- Sampling:

Purposive sampling was used, supplemented by snowball sampling.

- Method of approach:

Participants were approached through NGOs, community outreach, and referrals.

- Sample size:

76 participants: 41 AGYW (IDIs), 6 FGDs with AGYW, and 15 KIIs.

- Non-participation:

6 AGYW declined participation due to scheduling conflicts or consent refusal.

### Setting

- Setting of data collection:

Interviews and FGDs were conducted in NGO offices, community centers, or participants’ homes.

- Presence of non-participants:

No non-participants were present during data collection.

- Description of sample:

Demographic characteristics (age, education, marital status, etc.) are reported in the findings and methods sections.

### Data Collection

- Interview guide:

Semi-structured guides were piloted and contextually adapted.

- Repeat interviews:

None conducted.

- Audio/visual recording:

All sessions were audio-recorded with participant consent.

- Field notes:

Researchers made field notes during and after interviews.

- Duration:

Interviews lasted 45–90 minutes; FGDs lasted about 1.5–2 hours.

- Data saturation:

Data collection continued until no new themes emerged across sites and subgroups.

- Transcripts returned:

Not returned to participants due to confidentiality and literacy concerns.

## Domain 3: Analysis and Findings

### Data Analysis

- Number of data coders:

Three researchers independently coded the data and compared interpretations.

- Description of the coding tree:

A coding tree is described in the methods section.

- Derivation of themes:

Both inductive and deductive approaches were used.

- Software:

NVivo 12 was used for data management and coding.

- Participant checking:

Findings were validated through internal peer debriefings and member reflections via community partners.

### Reporting

- Quotations presented:

Yes, participant quotations are included to illustrate themes and attributed by participant type and location.

- Data and findings consistent:

Themes are grounded in participant narratives and consistent with the data.

- Clarity of major themes:

Major themes are clearly presented in the results.

- Clarity of minor themes:

Diverse or minor perspectives are also discussed to enrich the analysis.
